# Improving access to eye care for women and girls: what are the key areas for action?

**Published:** 2025-03-07

**Authors:** Clare Szalay Timbo, Preeti Dhingra, Jenni Pitter-López

**Affiliations:** 1 Technical Advisor, Gender and Special Projects: Orbis Canada, Seattle, USA.; 2 Head of Sustainability: Mission for Vision, Mumbai, India.; 3 Gender and Climate Expert: Light for the World, Vienna, Austria.

**Action is needed in several areas to overcome the unique barriers faced by women and girls, and gender-diverse people.**

**Figure:**
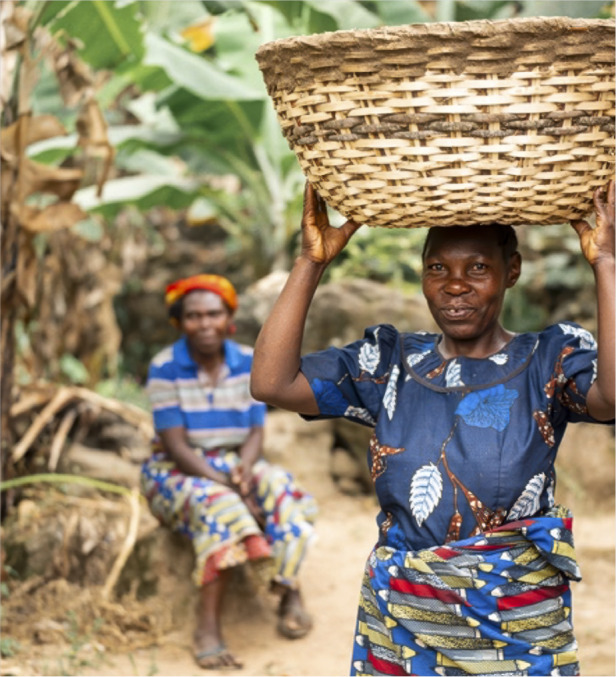
Nyibansabimana received sight-restoring cataract surgery after a community health worker referred her to an eye camp. rwanda

© FRED HOLLOWS FOUNDATION CC BY-NC-SA 4.0

It is only by collaboratively addressing the systemic barriers that impede equity of access to eye care for women, girls, and gender-diverse people that we can foster a more equitable eye health environment. To do this we need to focus on several areas of action.

## 1. Data, monitoring, and evaluation

Accurate data collection, evidence, and research are crucial for shaping future eye care initiatives. Comprehensive data (including sex/gender disaggregation) allows for the identification of gaps and the evaluation of health care programmes and treatment effectiveness.^1^

Evaluating the effectiveness of eye care programmes requires robust key performance indicators (KPIs) to monitor impact. Knowledge, attitudes, and practices (KAPs) assessments can be carried out to gauge community awareness and behaviours regarding eye health. Gender indicators are essential for tracking disparities in access and outcomes between different genders and ensuring that programmes address specific needs effectively. Satisfaction surveys can help to measure the quality of care and identify areas for improvement based on patient feedback.

More research is needed to explore gender inequities in eye care access and to develop targeted solutions that address these disparities. By continuously gathering and analysing data, stakeholders can refine strategies, enhance programme design, and implement evidence-based practices to improve eye care services and ensure equitable access for all. Research and sex/gender disaggregated data showing eye health disparities, quality of care differences, and gender access inequities can make a compelling case for policies and other targeted interventions.

## 2. Policy interventions

Policies that prioritise women's eye health are crucial for addressing disparities and improving outcomes.

Policy interventions can range from integrating eye health into broader health services specifically provided for women, such as maternal and child health services and sexual and reproductive health services, to ensuring that gender-specific needs are considered in national health strategies. Effective policies should include regular screening, affordable treatment, and public education campaigns that highlight the importance of eye health for women and girls. Read more in the UN Women report: bit.ly/UNwomeneye

Engaging policymakers and stakeholders in discussions about the unique challenges women face can help to create targeted and effective policies – including policies to increase the proportion of women represented in decision-making bodies and organisations.

**UN Women report**

No Woman Left Behind: Closing the Gender Gap in Eye Health

bit.ly/UNWeye

**Figure:**
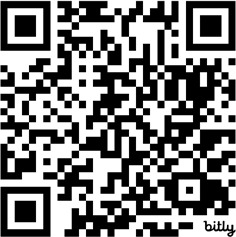

## 3. Interventions at the level of eye health programmes

Eye health programmes and initiatives that bring eye care closer to communities, especially rural communities, play a major role in improving access for women and girls, as transport and travel are significant barriers.

**Vision centres** are an example of a sustainable model of eye care provision for rural, underserved, and/or low-resource settings, particularly those that are densely populated, e.g. in South Asia.^2^ Vision centres can provide primary care, screening, and referral to a local hospital when needed. Vision centres staffed by women have been found to encourage greater uptake of eye health services among women.^3^

In many regions, **community health workers** have been trained to conduct eye screening and provide basic eye care, or to refer people to local hospitals or **outreach camps**, which significantly improves access to services for women and girls in remote areas. Examples are the National Lady Health Worker Programme in Pakistan and the female Community Health Workers in Ethiopia.

Training health workers in gender equality and equity, and in how to investigate inequities in access and decision-making locally, can also help to ensure cultural and gender barriers are overcome.^4^ These initiatives not only enhance access to care but also empower local communities to take ownership of their health.^5^

**From the field**

**Telemedicine in a woman-led vision centre**

**Figure:**
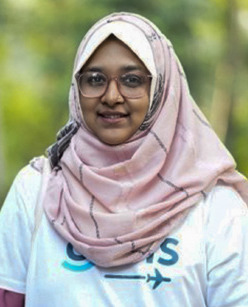
© ORBIS ZAMBIA CC BY-NC-SA 4.0

**Tasmia Tasmin Tani** is the allied ophthalmic practitioner in charge of Haimchar Vision Centre in Chandpur, Bangladesh. She is responsible for providing eye screening and primary eye care. “For patients requiring advanced consultation, we arrange tele-consultancy sessions with doctors at our base hospital.” Patients with severe eye conditions are referred to the base hospital.

“Women-led vision centres are important because people in these rural areas are not comfortable speaking openly with a man. But they can say everything in detail to a woman. These open discussions help provide patients with proper treatment.”

## 4. Collaboration and partnerships

Effectively addressing the eye health of women and girls requires collaboration. Multi-sectoral partnerships involving health care providers, NGOs, and community-led organisations (including women's or gender equity organisations) are essential for creating a comprehensive and inclusive approach. Such collaborations can lead to the development of innovative solutions and a more efficient use of resources.

Linking eye care with broader health and education initiatives ensures better integration and scalability. Collaborating with school health programmes and educational campaigns raises awareness and promotes early detection of vision problems. Aligning eye care with general health programmes supports a comprehensive approach to wellbeing, addressing immediate needs and fostering long-term improvements in eye health. For example, eye care can be provided in conjunction with other essential health services, such as maternal and child health, sexual and reproductive health, and child immunisations. This can be delivered via mobile or outreach services, or at community health centres.

## 5. Awareness and education

Engaging men and boys as advocates for women's eye health is also crucial. By involving them in awareness raising campaigns and educational programmes, eye care teams can challenge and change cultural norms that may undermine women and girl's health.

Awareness campaigns and educational programmes can also address issues facing women and girls as patients, such as stigma, bias, lack of access, or discrimination.

Educational campaigns are also needed to address the challenges facing women as health professionals, such as burnout, lack of supervision or adequate support, and pay gaps – while also considering their roles as care providers. This is vital for ensuring equitable and effective health care delivery.^6^

## 6. Technology

Telemedicine is revolutionising access to eye care, particularly for historically underserved populations. The vision centre model uses telemedicine to connect remote communities with specialised eye care through virtual consultations and remote diagnostics.^2^ This approach bridges geographical gaps and enhances the efficiency of services.

Innovations in eye care technology can help to improve early detection and treatment at the primary care level. Mobile apps such as Peek Acuity (bit.ly/41iFFL8) can enable anyone to check visual acuity. AI-powered tools can analyse retinal images for conditions like diabetic retinopathy and glaucoma, enhancing early intervention and care^7^ for female patients, who may face barriers to accessing traditional services.

In conclusion, it is imperative for everyone – including governments, health organisations, NGOs, and community leaders – to prioritise initiatives that directly address gender inequities. By investing in community health worker programmes, enhancing education about eye health, collecting sex disaggregated data, and expanding service availability through technology innovations, eye care teams can bridge the gap in eye care access and address gender inequities. Best practices also demonstrate that using gender equity tools, such as those shared in the IAPB Gender Equity Toolkit (bit.ly/IAPBgender), allow relevant stakeholders to effectively integrate gender into eye health planning and programming.

**From the field**

**Data, awareness campaigns, and cross-sectoral collaboration**

**Figure:**
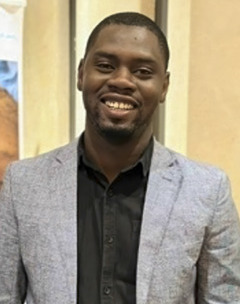
© ORBIS ZAMBIA CC BY-NC-SA 4.0

Monitoring and evaluation can provide helpful insights for programmes aiming to reduce disparities in eye health.

“Integrating gender into our monitoring and evaluation strategy has helped to make our programs more inclusive,” says **Enock Nsokolo**, Monitoring and Evaluation Manager at Orbis Zambia. “By gathering sex/gender-disaggregated data, we identified significant disparities in care access between men and women. For instance, we noticed that – while women visit eye health facilities more frequently than men – they are less likely to make use of surgical services.”

This understanding led to targeted interventions to close the gap, including community engagement and awareness campaigns and community outreach programmes in collaboration with mother and child health services.

“For eye health teams, our advice is to prioritise gender-disaggregated data collection. It's not just about numbers; it's about recognising and addressing the unique barriers that hinder equitable access to eye care for all and addressing them.”
